# Cross-sensory modulation in a future top predator, the young Nile crocodile

**DOI:** 10.1098/rsos.170386

**Published:** 2017-06-21

**Authors:** Laura Chabrolles, Gérard Coureaud, Nicolas Boyer, Nicolas Mathevon, Marilyn Beauchaud

**Affiliations:** 1 Université de Lyon/Saint-Etienne, Equipe Neuro-Ethologie Sensorielle, ENES/Neuro-PSI CNRS UMR 9197, Saint-Etienne, France; 2 Centre de Recherche en Neurosciences de Lyon, INSERM U1028/CNRS UMR 5292/Université Claude Bernard Lyon 1, Lyon, France

**Keywords:** acoustic, olfaction, cross-sensory modulation, behaviour, prandial state, playback

## Abstract

Animals routinely receive information through different sensory channels, and inputs from a modality may modulate the perception and behavioural reaction to others. In spite of their potential adaptive value, the behavioural correlates of this cross-sensory modulation have been poorly investigated. Due to their predator life, crocodilians deal with decisional conflicts emerging from concurrent stimuli. By testing young *Crocodylus niloticus* with sounds in the absence or presence of chemical stimuli, we show that (i) the prandial (feeding) state modulates the responsiveness of the animal to a congruent, i.e. food-related olfactory stimulus, (ii) the prandial state alters the responsiveness to an incongruent (independent of food) sound, (iii) fasted, but not sated, crocodiles display selective attention to socially relevant sounds over noise in presence of food odour. Cross-sensory modulation thus appears functional in young Nile crocodiles. It may contribute to decision making in the wild, when juveniles use it to interact acoustically when foraging.

## 1. Introduction

As animal behaviour is commonly driven by information coming from different sensory channels, internal conflicts about the appropriate behavioural response regularly arise from concurrent biological stimuli. Inputs from a given sensory modality can thus modulate the perception and behavioural reaction to another stimulus [1–3]. For instance, olfaction has been shown to be modulated by vision [4,5] and sounds [6]. Although probably widespread, this cross-sensory modulation of perception has mainly been studied through sensory analysis, psychological and neurobiological approaches, but paying no or weak attention to its behavioural and adaptive correlates [7–11]. As crocodilians routinely experience simultaneous stimuli from distinct sensory channels—chemical, acoustical, visual—from an early age, they have to deal with decisional conflicts emerging from concurrent stimuli, with crucial advantages in integrating various sensory cues to interact with congeners and improve prey localization and capture [12]. Using an experimental paradigm with young Nile crocodiles *Crocodylus niloticus* allowing testing of their behavioural responsiveness to food odour and/or social and non-social sounds, here we highlight a cross-modulation effect between olfactory and acoustic inputs. Strikingly, this cross-modulation varies according to an important regulating factor of motivation, the prandial state of the animals.

## 2. Material and methods

### 2.1. Animals and housing conditions

The study was conducted on 30 Nile crocodile juveniles (7–10 months old, undetermined sex, size: 33–41 cm), provided by the zoo ‘La Ferme aux Crocodiles' (Pierrelatte, France). The experiments started after three weeks of acclimatization in the laboratory. The crocodiles were separated in two groups and kept in rest tanks (180 × 120 × 50 cm) under a 12 : 12 light : dark cycle. Since these animals are mostly nocturnal, their circadian cycle was reversed gradually during the first 3 days post-arrival to allow performing experiments during daytime. A low intensity red light allowed the monitoring of animals during the nocturnal phase. Air and water temperatures were maintained at 26 and 28°C respectively. Each animal was identified by a white mark drawn on its skin. Animals were fed twice a week, at the end of the diurnal phase, with chicken pieces (2–3 g/individual/feeding).

### 2.2. Acoustic stimuli

Contact calls of four different young Nile crocodiles (4–6 days old) were used for the playback experiments. These calls had been previously recorded in the laboratory from unfamiliar individuals, which were released into a tank where they could meet other siblings for the first time. In this situation, young crocodiles use contact calls to communicate [13,14].

An acoustic stimulus consisted of a series of repeated contact calls from the same individual (three renditions of the same call emitted in 2 s, which corresponds to a natural emission rhythm). During a test, a series was repeated nine times. These renditions were separated by silence periods of variable durations (1 s, 1 min or 5 min) to mimic natural sequences of acoustic interactions (detail of these durations: 1 s between the first and second call series, 1 min between the second and the third, 1 s between the third and the fourth, 5 min between the fourth and the fifth, and then 1 s between the remaining four series). To limit pseudo-replication, each tested individual was challenged with contact calls coming randomly from one of the four recorded crocodiles.

A white noise was used as control (bandwidth = 100–5000 Hz). The control sequence was organized identically to the call playback, except that the series of three successive calls was replaced by a continuous 2 s white noise to avoid the rhythmic effect produced by the alternation of calls and short silences.

### 2.3. Double-choice set-up

**Figure 1.**
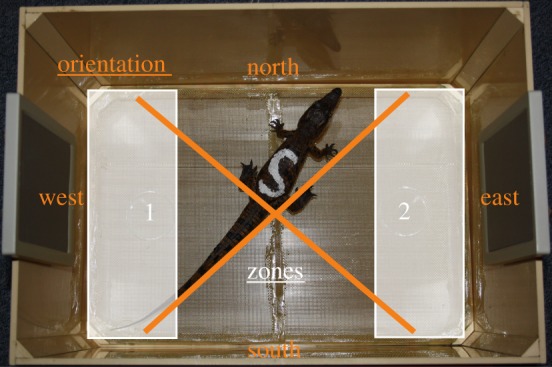
Experimental arena divided into two zones (1 and 2) and four orientation areas (north, south, east and west). An active loudspeaker was set up on one side, while there was either water or meat on the other side. To avoid spatial bias, both sides were permuted between tested individuals. Cardinal points were used to identify the animal's head orientation.

The behavioural tests were run in a rectangular arena (50 × 30 × 30 cm) with a wire-mesh platform (41 × 28 × 2 cm; mesh size: 1 mm^2^) divided in half by a median line. Below the platform, two Petri dishes (diameter: 4.7 cm) were placed at equal distance (2.5 cm) from each of the extremities, which delimited two zones containing the Petri dishes (figure 1; see [15,16] for an initial use of such apparatus in young rabbits). The Petri dishes contained either water or meat. This experimental set-up was located in a sound-proofed chamber (PRIMO Silence-Box, Tip Top Wood©). The temperature and luminance of the chamber were the same as for the housing room. Two loudspeakers (Kinyo, SW 201, Taiwan, frequency response : 80–16 000 Hz) were settled in each of the extremities of the arena. During experiments with sounds, only one of the two loudspeakers was used for playback. The active loudspeaker was changed alternatively between tests. PRAAT software [17] was used to control the playback of the acoustic stimuli.

Behavioural answers were recorded using a camera (BUL520, Active Media Concept, Vallauris, France) suspended above the arena and connected to a computer located outside the sound-proofed chamber. Just before releasing a crocodile into the arena, the experimenter started the video-monitoring. Each crocodile was introduced into the arena with its sagittal axis aligned on the midline of the platform and was left free over the wire platform. At the end of the experiment, the animal was reintroduced into its rest tank, and the arena rotated at 180° to limit bias due to potential chemical cues in the environment. Every three individuals, the arena and the wire mesh platform were rinsed with 100° ethyl alcohol and pure water and dried; meat and water were replaced by new samples. To control for side biases, we balanced the stimuli position between the left and the right sides. Each individual was tested with only one treatment per day, with 2–6 days between two consecutive tests. An individual was submitted to a minimum of three and a maximum of seven different assays.

### 2.4. Assessment of the individuals' feeding state

We conducted preliminary observations to determine how long animals remain sated after a feeding episode. We manually fed individuals (*n* = 13) until they refused the food during 10 consecutive minutes. Two other feeding sessions were proposed respectively 1 and 2 h after the first one. During the first feeding session, individuals ate between 5 and 14 g of chicken (median weight: 9.13 g). Only two individuals ate again during the second session, while only one accepted to be fed during the third session. For the experimental tests, we therefore considered that an individual remained ‘sated’ during the 2 h following a feeding session. ‘Fasted’ individuals were not fed during 2 consecutive days prior to an experimental test.

### 2.5. Experiments

In a first experiment, we tested whether juvenile crocodiles display differential locomotor activity in response to food odour depending on their prandial state. One of the Petri dishes of the double-choice arena contained water and the other one contained a piece of meat. No sound was emitted by the loudspeakers. We tested 13 ‘sated’ crocodiles and 10 ‘fasted’ crocodiles.

Then, in the second and third experiments, we did two different playback assays:

— Playback 1 (‘no food’ condition) challenged both fasted (*n* = 26) and sated (*n* = 24) individuals with juveniles' contact calls or white noise *in the absence of any odour source*. One of the loudspeakers emitted the sounds while water was present on the other side of the arena.
— Playback 2 (‘presence of food’ condition) challenged both fasted (*n* = 24) and sated (*n* = 24) individuals with juveniles' contact calls or white noise *in the presence of an odour source*. Here we thus assessed if the responsiveness of crocodiles to sound can be modulated by food odour, and whether this putative effect depends on both the nature of the sound (contact call versus noise) and the prandial state (sated versus fasted) of the animal. One of the loudspeakers emitted the played back sounds while a piece of food was present on the other side of the arena (figure 1).

### 2.6. Analysis of behavioural responses

We characterized the behavioural responses of crocodiles to the experimental stimuli by measuring two variables:

(1) the time (in seconds) spent in the two zones containing either meat or water (figure 1); an animal was considered to be in one zone when its nostrils and eyes were in that zone;
(2) the head orientation time (in seconds), displayed alone or in addition to other body movements. Each movement of the head or head + body was quantified according to the cardinal points (figure 1). For example, an individual was considered facing east if its head orientation was north-east, east or south-east. This parameter allowed us to determine the time spent oriented towards the acoustic stimulus (sound or noise) emitted by the loudspeaker (while the active loudspeaker was on the right (east) or on the left (west)).

When no sound was broadcasted, we assessed only the presence time in the two pre-defined zones of the arena. Behavioural responses were analysed using EthoLog v. 2.2 software [18].

### 2.7. Statistical analysis

Due to the limited size of the groups, non-parametric tests were used. When no sound was broadcasted, the mean presence time spent on the zones above food versus water was compared with non-parametric permutation t tests for paired data (‘perm.t.test’ function from ‘RVAideMemoire’ R package [19]). In Playback 1 ‘no food’ condition, and Playback 2 ‘presence of food’ condition, we compared the head orientation times and the presence times spent in a specific area of the arena in different manners depending on the variance's homogeneity (the variance's homogeneity was tested with a non-parametric permutation Fisher test, ‘perm.var.test’ function from ‘RVAideMemoire’ R package): when the variance was homogeneous, non-parametric permutation t tests were used for comparisons between individuals submitted to a given choice, while Mann–Whitney tests were used when the variance was not homogeneous. To compare the behaviour of same individuals in different conditions of choice tests, non-parametric permutation t tests for paired data were used. For all comparisons, statistical differences were considered significant when *p* ≤ 0.05 (without Bonferroni correction). Statistical tests were conducted using R v. 2.14.0 software [20].

## 3. Results and discussion

**Figure 2.**
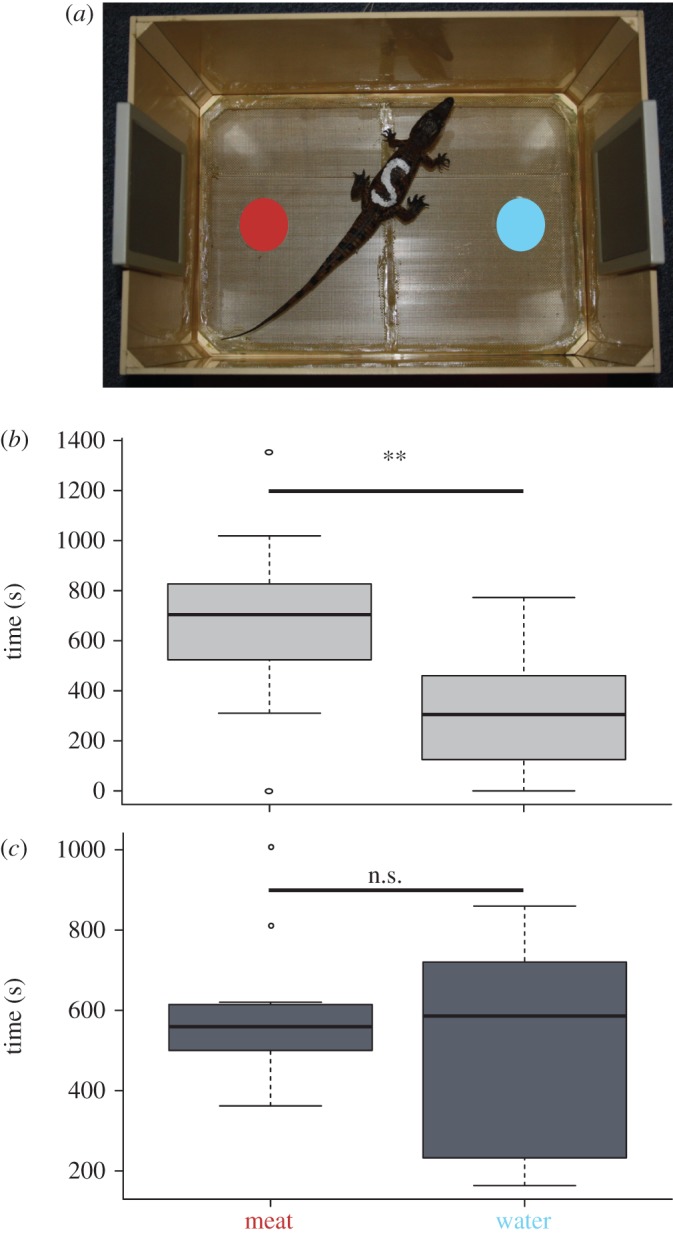
Young Nile crocodiles' preference to food odour versus water according to their prandial state. (*a*) Double-choice testing device (red dot: area with meat; blue dot: area with water). The boxplots show the presence time over each of the areas for (*b*) fasted (*n* = 10) and (*c*) sated (*n* = 13) crocodiles. n.s., non-significant difference; **p ≤ 0.01.

When challenged in the double-choice test with meat odour versus water (first experiment, without sound), fasted crocodiles were more attracted by the odour of meat (*n* = 10, *t* = −2.39, *p* = 0.01) while sated individuals did not display any preference between stimuli (*n* = 13, *t* = −0.62, *p* = 0.56; figure 2). In this experimental condition where no sound stimulus was emitted, the prandial state thus unsurprisingly modulates the responsiveness of young crocodiles to a congruent, i.e. food intake-related, olfactory stimulus.

**Figure 3.**
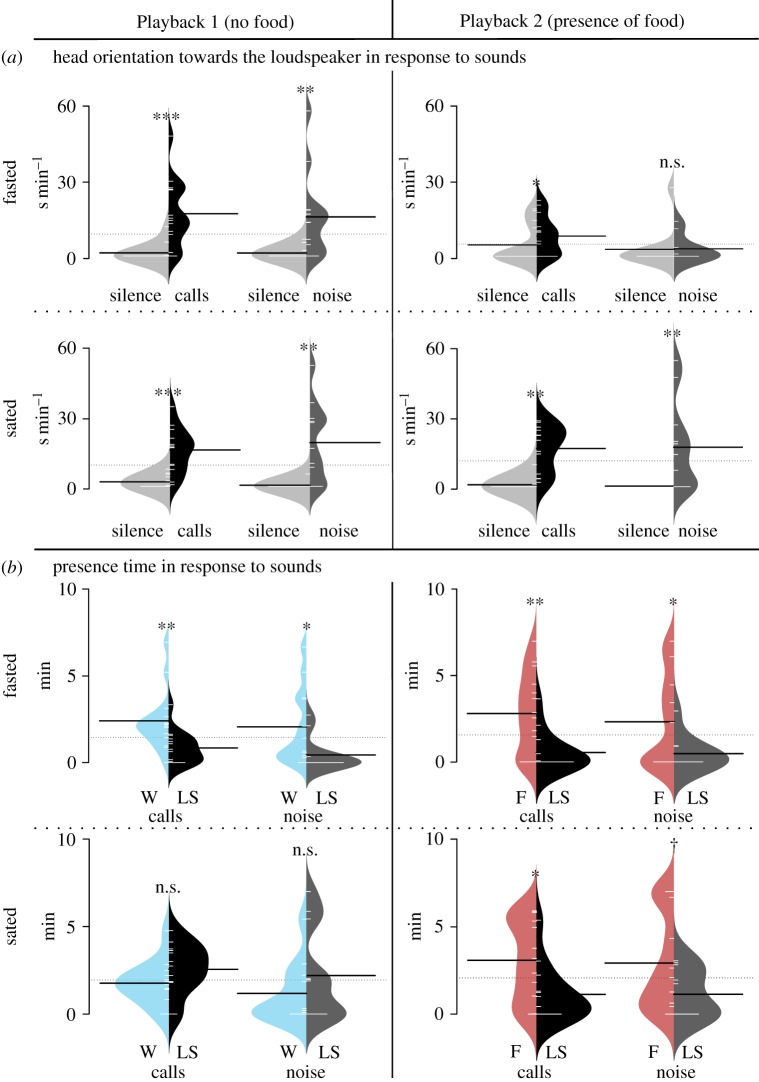
Behavioural response of young Nile crocodiles to sound stimuli according to their prandial state (fasted or sated) and the absence or presence of food. (*a*) The time spent oriented towards the loudspeaker (head orientation) was assessed during both the pre-playback period (silence) and the playback period (calls or noise). (*b*) The time spent close to the loudspeaker (LS) and the time spent at the opposite side (containing either water (W) or food (F)) were measured during the playback of sound signals (calls or noise). Beanplots (‘beanplot’ R package) combine individual observations (white horizontal lines), dataset distribution, the overall dataset average (dashed horizontal line) and the average for each subset (black horizontal lines). n.s., non-significant, ^†^*p* < 0.1, *p ≤ 0.05, **p ≤ 0.01, ***p ≤ 0.001.

However, the first playback assay (Playback 1), in absence of any odour (food) source but with a sound stimulus, further showed that the feeding state of juvenile crocodiles also alters their responsiveness to an incongruent—i.e. independent of food—stimulus. Although all tested crocodiles oriented their head towards both sound stimuli during the playback compared to the silent pre-playback period (fasted animals challenged with calls, *n* = 14, or noise, *n* = 12: *t* = 3.86 or 2.84, *p* < 0.01; sated individuals challenged with calls, *n* = 13, or noise, *n* = 11: *t* = 5.57 or 3.52, *p* < 0.01; Playback 1, figure 3*a*), only sated individuals explored equivalently both sides of the arena—including the sound side—during playback (time spent by sated individuals on opposite versus loudspeaker side, for calls or noise respectively: *t* = −1.45 or −0.90, *p* = 0.18 or 0.43; Playback 1, figure 3*b*). Conversely, fasted individuals stayed preferentially on the water side (*t* = 2.80 or 2.25, *p* < 0.01 or 0.02; Playback 1, figure 3*b*). Thus, in the absence of food odour, both fasted and sated crocodiles display attention (head orientation) to social and non-social sounds, but these stimuli fail in eliciting a locomotor response in fasted individuals.

Finally, the second playback assay (Playback 2, ‘presence of food’ condition) directly investigated our main question, i.e. how the olfactory and auditory channels interact during behavioural decisions. When meat odour was opposed to sound, fasted and sated animals mainly explored the food side compared to the loudspeaker side of the arena, whatever the sound (time spent by fasted individuals on food odour versus loudspeaker side, for calls or noise respectively: *n* = 14 or 10, *t* = 3.14 or 1.89, *p* < 0.01 or *p* = 0.05; for sated individuals: *n* = 13 or 11, *t* = 2.08 or 1.67, *p* = 0.03 or 0.07; Playback 2, figure 3*b*). However, although fasted crocodiles still oriented their head towards the loudspeaker in response to calls (*n* = 14, *t* = 1.56, *p* = 0.05; Playback 2, figure 3*a*), the noise stimulus was no longer efficient to elicit such head orientation (*n* = 10, *t* = 0.05, *p* = 0.56; Playback 2, figure 3*a*). Conversely, sated individuals oriented their head significantly towards the loudspeaker in response to both playback signals (calls: *n* = 13, *t* = 5.95, *p* < 0.01; noise: *n* = 11, *t* = 2.94, *p* = 0.01; Playback 2, figure 3*a*). Thus, young crocodiles prioritize food over sound stimuli whatever their prandial state and the nature of sound, but when fasted they display selective attention to socially relevant acoustic signals over noise in presence of food odour.

In summary, the prandial state influences behavioural responsiveness to food odours but also to sounds in juvenile crocodilians. In addition, and strikingly, the odour of food modulates their response to social calls, i.e. to signals from the acoustic modality. This cross-sensory modulation varies according to the prandial state of the animal, therefore to physiological processes related to motivation. Thus, young Nile crocodiles, which are known to gather together in pods and to strongly depend on their mother for protection [12], may already benefit from prioritizing distinct environmental sensory cues including intraspecific sounds, to maintain cohesion between siblings, interaction with the mother, and to be alerted for the presence of food. It is also noticeable that young crocodilians have been observed producing contact calls when being fed or in the presence of food [21]. This may explain why, in our study, fasted individuals—i.e. young animals likely to search for food—responded selectively to this variety of calls compared to noise. Furthermore, at the adult age, they could use this ability in a multimodal sensory context including also interspecific sounds emitted by prey. Further research is required to determine how such cross-sensory modulation evolves during development and what are the physiological processes involved in its expression.

## Supplementary Material

ES1

## Supplementary Material

ES2

## Supplementary Material

ES3

## Data accessibility

Raw data are included in the electronic supplementary material.
